# Aztreonam-avibactam synergy, a validation and comparison of diagnostic tools

**DOI:** 10.3389/fmicb.2023.1322180

**Published:** 2023-11-29

**Authors:** Gil Verschelden, Maxim Noeparast, Anke Stoefs, Eveline Van Honacker, Kristof Vandoorslaer, Laura Vandervore, Margo Olbrecht, Kathleen Van Damme, Thomas Demuyser, Denis Piérard, Ingrid Wybo

**Affiliations:** ^1^Department of Internal Medicine and Infectious Diseases, Universitair Ziekenhuis Brussel (UZ Brussel), Brussels, Belgium; ^2^Department of Microbiology and Infection Control, Universitair Ziekenhuis Brussel (UZ Brussel), Brussels, Belgium; ^3^Translational Oncology, University Medical Center Augsburg, Augsburg, Germany; ^4^AIMS Lab, Center for Neurosciences, Faculty of Medicine and Pharmacy, Vrije Universiteit Brussel (VUB), Brussels, Belgium

**Keywords:** aztreonam, avibactam, ceftazidime/avibactam, *Enterobacterales*, *P. aeruginosa*, *S. maltophilia*, antimicrobial resistance, antibiotic synergy

## Abstract

**Introduction:**

Antimicrobial resistance is a growing problem that necessitates the development of new therapeutic options. Cefiderocol and aztreonam (AT) are often the last active β-lactams for treating metallo-β-lactamases (MBL)-producing Gram-negative bacilli. In these difficult-to-treat bacterial strains, AT resistance is frequently attributed to the co-occurrence of other resistance mechanisms. In the case of β-lactamases they can often be inhibited by avibactam. In the present study, we evaluated the use of the double-disc synergy test (DDST) as a screening tool for the detection of synergy between AT-avibactam (ATA). We validated both the Gradient Diffusion Strips (GDSs) superposition method and the commercially available Liofilchem’s ATA GDS.

**Materials and methods:**

We tested AT susceptibility in combination with ceftazidime-avibactam for 65 strains, including 18 Serine-β-Lactamase (SBL)- and 24 MBL-producing *Enterobacterales*, 12 MBL-producing *P. aeruginosa*, and 11 *S. maltophilia* isolates. Interpretation was done with EUCAST breakpoints (version 13.0), AT breakpoints being used for ATA. The accuracy and validity of the GDSs superposition method and ATA GDS were evaluated using an AT GDS applied on Mueller Hinton Agar plates supplemented with avibactam (MH-AV). A DDST was performed to screen for synergy between antibiotic combinations.

**Results:**

Using MH-AV, all SBL- and MBL-positive *Enterobacterales* were susceptible or susceptible at increased exposure to the combination AT-avibactam. In contrast, only 2 out of the 12 (17%) *P. aeruginosa* strains and 9/11 (82%) of the *S. maltophilia* strains were susceptible- or susceptible at increased exposure for the combination of AT-avibactam. The DDST detected all synergies, demonstrating a 100% sensitivity and 100% negative predictive value for all bacterial strains.

**Conclusion:**

The DDST is a sensitive tool for screening for antibiotic synergy. Unlike *S. maltophilia* and SBL- and MBL-positive *Enterobacterales*, most MBL-positive *P. aeruginosa* strains remain resistant to AT-avibactam. ATA GDS should be preferred for MIC determination of the AT-avibactam combination, while the GDSs superposition method can be used as an alternative to the commercial test.

## Introduction

Antimicrobial resistance is a growing concern and has been identified by the European Commission as one of the top 3 priority health threats in July 2022. The World Health Organization (WHO) has also recognized it as one of the top 10 global public health threats facing humanity in 2019 (Akbar, 2019; Health Emergency Preparedness and Response Authority, 2022). In 2019, it was estimated that 1.27 million deaths worldwide were directly attributed to antibiotic-resistant infections. Among resistance-related deaths, *E. coli* is the leading pathogen, followed by *S. aureus*, *K. pneumoniae*, *S. pneumoniae*, *A. baumannii*, and finally *P. aeruginosa* (Murray et al., 2022).

The most common and major resistance mechanism is the degradation of β-lactam antibiotics by hydrolysis, which was first described in 1940 (Hall and Barlow, 2004). The β-lactamases produced by bacteria catalyze the hydrolysis of β-lactams, hindering the acetylation and, therefore, making penicillin-binding protein (PBP) inhibition impossible. The Ambler classification system of β-lactamases, distinguishes 4 groups according to their enzymatic structure. Class A, C and D all contain a serine residue in the active site (Serine-β-Lactamase, SBL). In contrast, class B belongs to the Metallo-β-Lactamases (MBL), which confer their activity thanks to one or two zinc^2+^ ions in their active site making them resistant to 4^th^-generation cephalosoprins and carbapenems (Bush, 2018).

The prevalence of Carbapenem Resistant *Enterobacterales* (CRE) has risen steadily since the early 1990s, first described in Europe, and now reaching global proportions (Brolund et al., 2019; Nordmann and Poirel, 2019). Epidemiology in Europe varies considerably nowadays, with a strong north–south gradient. Ranging from sporadic imported cases of carbapenem-resistant *K. pneumoniae* in the Netherlands (0.2% in 2021) to a situation such as in Greece, where hospital-related CRE infections have become an endemic problem (73.7% in 2021), threatening not only the affected patient (increased mortality) but also the national economic system (Antimicrobial resistance surveillance in Europe 2023–2021 data, 2023).

The growing resistance of Gram-negative bacilli has therefore stimulated the development of new antibiotics and novel combinations of β-lactamase and β-lactamase-inhibitors. Ceftazidime-avibactam (CZA), Food and Drug Administration approved since February 2015, is an example of such combinations. Avibactam covalently binds to the serine residue of β-lactamase. Unlike clavulanic acid and tazobactam the molecule is not hydrolyzed, it slowly dissociates, and returns to its original structure to inhibit a new β-lactamase. Avibactam thus recovers the activity of ceftazidime (third-generation cephalosporin) in class A (ESBLs, KPCs), class C (AMPc), and class D (OXA-48) β-Lactamases (Falcone and Paterson, 2016). However, avibactam (like all β-lactamase inhibitors) remains inactive against MBLs.

On the other hand, MBLs and OXA-48 s, unlike KPCs, have little or no binding capacity to aztreonam (AT), thereby preventing its hydrolysis. Avibactam in combination with AT is therefore valuable for MBL strains that have lost their susceptibility to AT due to a chromosomal AMPc derepression (overexpression), or the acquisition of a plasmid mediated ESBL (CTX-M-type, SHV-type, TEM-type), AMPc-type (CMY-2), or KPC-type (Ruppé et al., 2015).

*S. maltophilia* is another well-known target for the application of the combination of β-Lactam and a β-lactamase inhibitor. The combination of two intrinsic and inducible β-lactamases, L1 and L2, confers natural resistance to all β-lactam antibiotics.

L1 is an MBL (Ambler class B) that confers resistance to all β-lactams (including β-lactamase inhibitors), except AT. L2, on the other hand, is a clavulanic acid sensitive β-lactamase (Ambler class A) hydrolyzing most β-lactams, including 2nd and 3rd generation cephalosporins and AT. Co-administration of a β-lactamase inhibitor that inhibits L2 may prevent hydrolysis of AT and restore its activity against L1 (Calvopiña et al., 2017).

It is therefore essential to assess the susceptibility of these bacterial strains for the AT-avibactam (ATA) combination. In the absence of ATA Gradient Diffusion Strips (GDSs), various methods have been proposed to test the susceptibility of bacterial strains. Either by sequential application of CZA and AT GDSs on a Mueller-Hinton Agar (MHA) plate, strip stacking or by crossing the GDSs (Emeraud et al., 2019; Khan et al., 2021).

Although accessible, these methods requires the use of 2 strips, either simultaneously or successively, depending on the chosen procedure. Apart from increasing the cost and workload, this procedure requires additional handling, which can lead to imprecisions or bacterial contaminations.

To answer this question, we validated both the GDSs superposition method and commercially available Liofilchem’s ATA GDSs, comparing the obtained results with those obtained using an AT GDS applied on an in-house Mueller Hinton Agar plates supplemented with avibactam (MH-AV), 4 mg/L.

We also evaluated the use of the Double Disk Synergy Test (DDST) as a screening tool for the detection of synergy between AT and avibactam, which could be useful in the selection of GDSs in a resource-saving manner (Falcone et al., 2021).

## Methods

### Bacterial isolates and susceptibility testing

Sixty-five bacterial strains were selected from a collection of multidrug-resistant microorganisms (MDRO) including 378 *Enterobacterales*, 2,191 *P. aeruginosa*, and 1,118 *S. maltophilia*, stored at −80°C in Mueller-Hinton broth with 20% glycerol between January 2010 and Mai 2023 in a tertiary university hospital (UZ-Brussel).

First, 18 SBL (KPC and/or OXA48) producing *Enterobacterales* were selected, all AT and meropenem resistant but CZA sensitive.

Thirty-six AT and CZA resistant MBL-producing bacterial strains were selected, among which 12 *P. aeruginosa* (VIM) and 24 *Enterobacterales*. These 24 MBL-producing *Enterobacterales* (all NDM) included 14 *K. pneumoniae*, 6 *E. coli*, 2 *C. freundii*, and 2 *E. cloacae*.

Finally, 11 *S. maltophilia* strains were selected. All selected strains were resistant to Sulfamethoxazole-Trimethoprim, AT and CZA.

Each strain was transferred to 5% sheep blood agar plates prior to testing.

Matrix-assisted laser desorption time-of-flight mass spectrometry (MALDI-TOF, Bruker Daltonics, Brussels, Belgium) was used for pathogen identification.

Antimicrobial susceptibility testing and determination of minimum inhibitory concentrations (MICs) were performed using a Sensititre™ system (Thermo Fisher Scientific®, Merelbeke, Belgium) with a 0.5 McFarland suspension according to the European Committee for Antimicrobial Susceptibility Testing (EUCAST) guidelines (version 13.0; The European Committee on Antimicrobial Susceptibility Testing, 2023a,b).

EUCAST breakpoints were considered for the interpretation of the susceptibility of bacterial strains based on their MICs (Table 1).

**Table 1:** MIC breakpoints for antimicrobials according to EUCAST guidelines.

Antimicrobial(s)	MIC (μg/ml) breakpoints^i^					
	*Enterobacterales*		*P. aeruginosa*		*S. maltophilia* ^ii^	
	S	R	S	R	S	R
Aztreonam (AT)	≤1	>4	≤0.001	>16	≤4	>8
Ceftazidime (CZ)	≤1	>4	≤0.001	>8	≤4	>8
Ceftazidime-avibactam (CZA)	≤8	>8	≤8	>8	≤8	>8

i Interpretations of susceptibility to CZA and AT-avibactam combination was based on CZA and AT EUCAST breakpoints, respectively, for the tested microorganism (European Committee on Antimicrobial Susceptibility Testing, version 13.0).

ii PK/PD breakpoints of AT were used to interpret the MIC for *S. maltophilia* as there is no species-specific recommendation for this antibiotic in this species.

Interpretations of susceptibility to ATA were based on a breakpoint of AT for the tested microorganism.

Pharmacokinetics/pharmacodynamics (PK/PD) breakpoints of AT and CZA were used to interpret the MIC for *S. maltophilia*, as there is no species-specific recommendation for these antibiotics in this pathogen.

*K. pneumoniae* ATCC 700603 strain was used for quality control of CZA GDS as proposed by EUCAST (The European Committee on Antimicrobial Susceptibility Testing, 2023a,b).

Finally, the presence of carbapenemases (SBL and/or MBL) was confirmed using a multiplex lateral flow immunochromatographic assay from CORIS BioConcept® (Gembloux, Belgium) for the detection of NDM, VIM, IMP, KPC and OXA-48.

### Synergy screening using a double-disc synergy test

**Figure 1:**
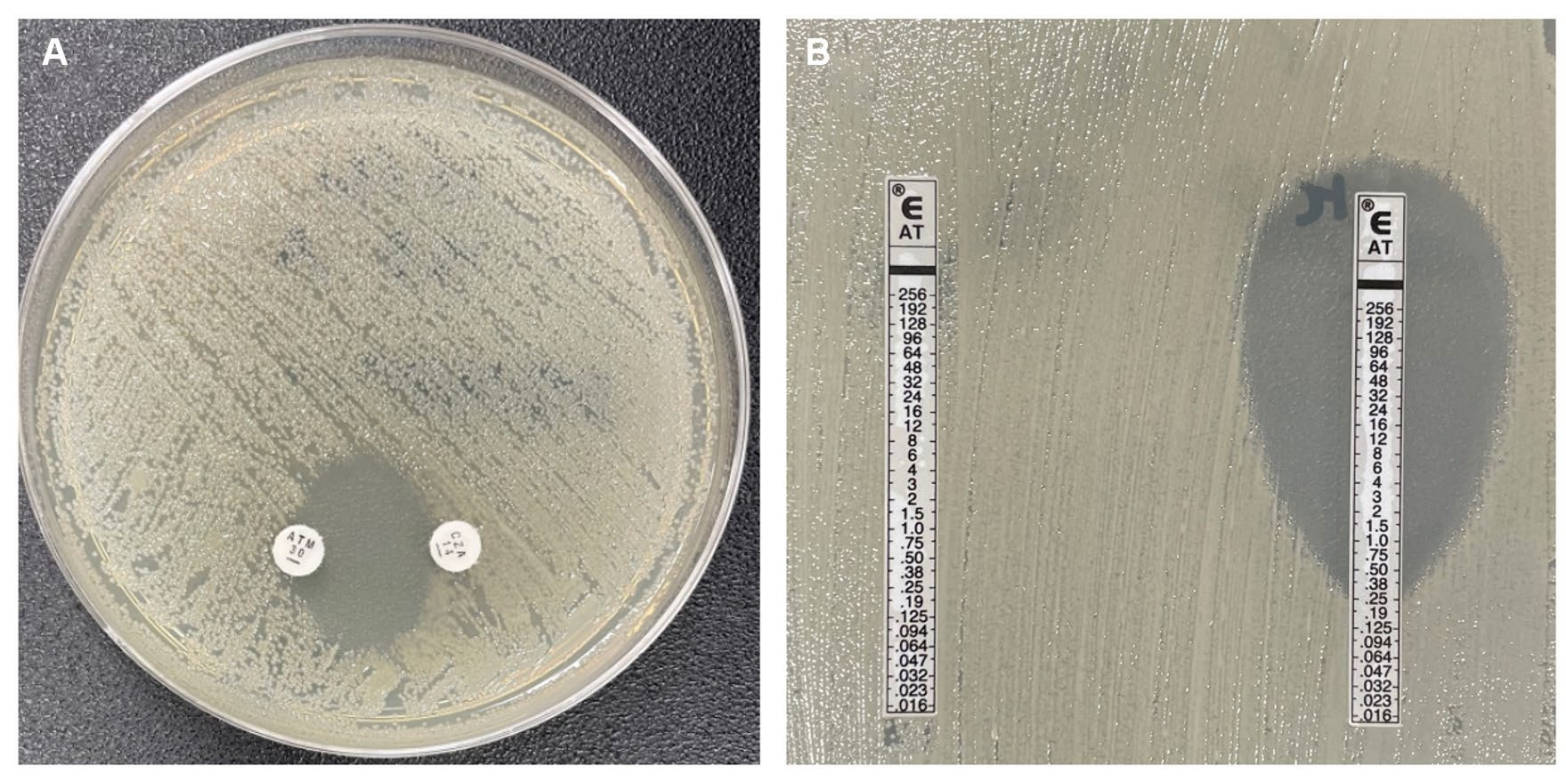
**(A)** Aztreonam (AT) and Ceftazidime-avibactam (CZA) double-disc synergy test. **(B)** Left: controle AT Gradient diffusion strip (GDS), Right: AT and CZA GDSs superposition methode.

The synergy between CZA and AT was screened using a double-disc diffusion method (Figure 1).

The CZA 14 μg and AT 30 μg discs from Oxoid® (Thermo Fisher Diagnostics®, Merelbeke, Belgium) were placed 20 mm apart (measured from the center of the disk), on a MHA plate.

The combination of two antibiotics was considered synergistic if an inhibition zone was observed between the two discs (Falcone et al., 2021).

The sensitivity of the method was subsequently assessed by comparing the obtained results with the gold standard, which we defined (in default of broth microdilution) as the restauration of the susceptibility or susceptibility at increased exposure for AT using an AT GDS applied on an in-house MH-AV. In the absence of an EUCAST recommendation for ATA sensitivity testing, the concentration of avibactam was fixed at 4 mg/L, as required by EUCAST for susceptibility testing of CZA (The European Committee on Antimicrobial Susceptibility Testing, 2023a,b).

### Synergy confirmation and determination of the MIC

The MIC of AT after avibactam supplementation was measured using two different methods (Figure 1):

First, using a GDSs superposition method: a CZA GDS from bioMérieux® (Schaarbeek, Belgium), containing a fixed concentration of avibactam 4 mg/L, was applied on MHA plates for 10 min at room temperature. The strip was subsequently replaced by an AT GDS to determine the MIC of the ATA combination (Emeraud et al., 2019).

Secondly, using commercially available ATA GDSs from Liofilchem® (ElitechGroup Benelux, Spankeren, The Netherlands).

MICs were measured after 16 h incubation at 35°C in ambient air and interpreted in accordance with EUCAST guidelines.

Synergy between AT and avibactam was interpreted as follows:

- Synergy ≥2 two-fold dilution decrease in MIC.
- Indifference <2 two-fold dilution decrease in MIC.

### Validation of the GDSs superposition method and the ATA GDS

The validity CZA-AT GDSs superposition method was evaluated by comparing the obtained MIC from multi-resistant *S. maltophilia*- and MBL-positive strains (*N* = 47), with that of an AT GDS applied to in-house MH-AV, 4 mg/L (gold standard).

The ATA GDSs, on the other hand, were validated using all 65 bacterial strains (including the SBL-producing *Enterobacterales* strains).

### Statistical methods

Data were analyzed using GraphPad Prism version 9.0.0 for Mac, GraphPad Software, San Diego, California USA, www.graphpad.com.

Shapiro–Wilk test with a significance value of >0.05 was used to assess the assumption of normality of MIC values.

MIC values are reported as the MIC50, MIC90, and MIC ranges.

Passing Bablok regression and Spearman’s correlation were used to compare MIC values of the commercial ATA GDS with the MIC of AT obtained on MH-AV.

The validation of the tests was confirmed when a Precision reproducibility Essential Agreement (PEA) of at least 95% was achieved (agreement within a single two-fold dilution compared to the results achieved using the MH-AV).

Finally, McNemar’s exact test was used to determine the significance of the difference in the occurrence of ±1 two-fold dilution between the use of the superposition method and commercial ATA GDS compared to the reference method.

## Results

### Synergy screening using a DDST

The DDST was able to detect all synergies in SBL- and MBL-producing *Enterobacterales* strains (Figure 1; Table 2), showing a sensitivity of 100% with a 95% confidence interval (CI) of (92 to 100%).

**Table 2:** MIC and susceptibility interpretation^i^ of CZA and ATA combinations on the different bacterial strains.

Bacterial strain		MIC (μg/ml)^i^			
	DDST	MIC 50	MIC 90	Range	Resistance rate
*S. maltophilia* (*n* = 11)					
ATA	Sens. 100 (71–100)	2	12	2 to 24	2/11 (18%)
	Spec. NA				1/11 (9%)^ii^
*P. aeruginosa* MBL (*n* = 12)					
ATA	Sens. 100 (29–100)	24	128	2 to 192	10/12 (83%)
	Spec. 78 (40–97)				
	NPV 100 (59–100)				
*Enterobacterales* (*n* = 42)					
ATA	Sens. 100 (92–100)				
	Spec. NA				
*Enterobacterales* MBL (*n* = 24)					
ATA		0.25	3	0.064 to 3	0/24
					5/24 (21%)^ii^
*Enterobacterales* SBL (*n* = 18)					
CZA		2	4	0,38 to 4	0/18
ATA		0.38	0.75	0.064 to 1.5	0/18
					1/18 (<0.1%)^ii^
					

AT: aztreonam, ATA: aztreonam/avibactam combination, CZA: ceftazidim-avibactam, DDST: double-disc synergy test, EUCAST: European Committee on Antimicrobial Susceptibility Testing, MH-AV: Mueller Hinton agar plate supplemented with avibactam, NA: not applicable, NPV: negative predictive value, Sens.: sensitivity, Spec.: specificity.

i MIC of the ATA combination obtained using a AT GDS applied on MH-AV 4 mg/L. Interpretations of susceptibility to CZA and ATA combination was based on CZA and AT EUCAST breakpoints (version 13.0) respectively for the tested microorganism. PK/PD breakpoints of AT were used to interpret the MIC for S. maltophilia as there is no species-specific recommendation for this antibiotic in this species.

ii Susceptible at increased exposure (I).

DDST also demonstrated its utility in the MBL-producing *P. aeruginosa* strains, where the method showed a sensitivity and specificity of, respectively, 100% (95% CI, 29 to 100%) and 78% (95% CI, 40 to 97%), with a Negative Predictive Value (NPV) of 100% (95% CI, 59 to 100%).

**Figure 2:**
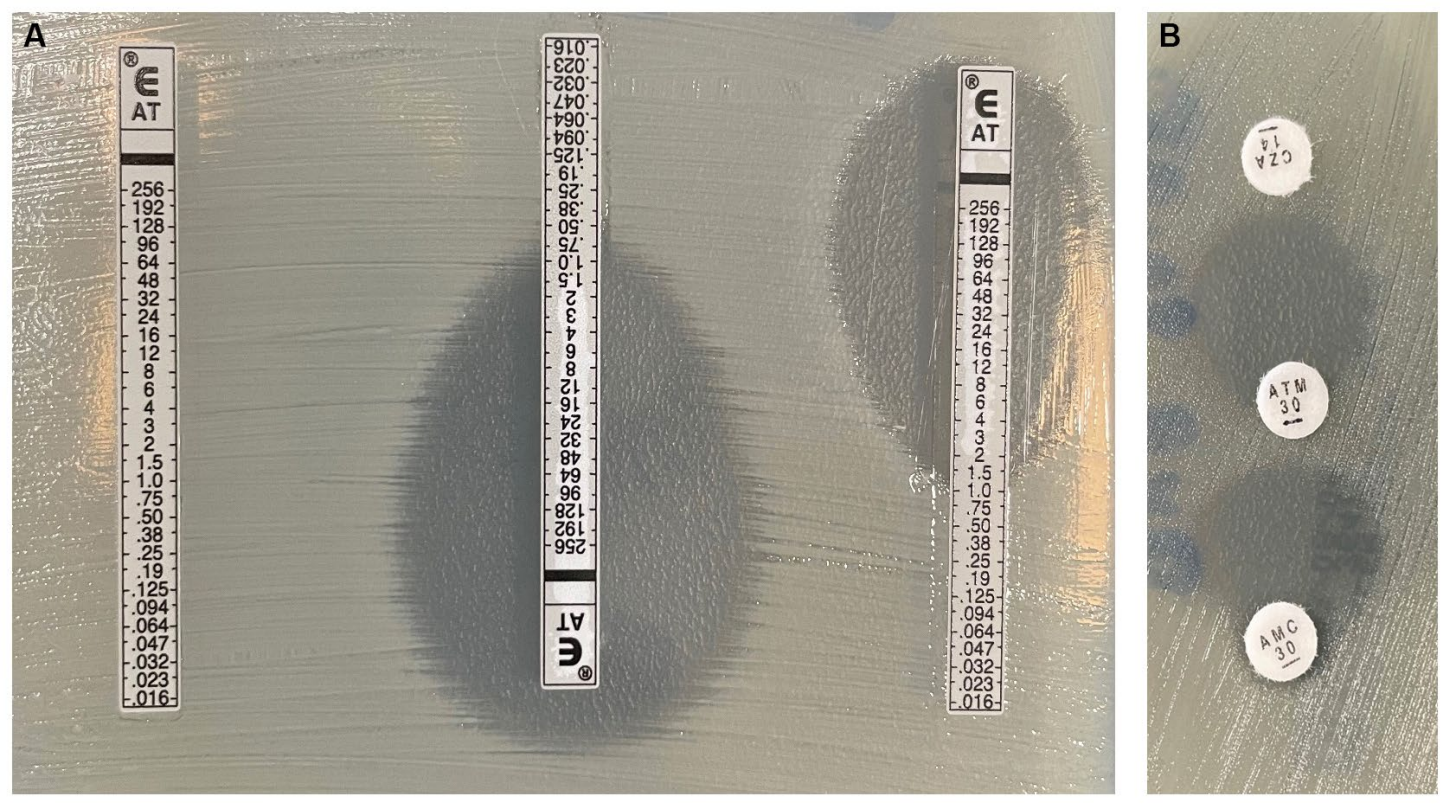
**(A)** Left: controle Aztreonam (AT) Gradient diffusion strip (GDS), Middle: AT and cefazidime-avibactam (CZA) GDSs superposition methode, Right: AT and amoxicillin clavulanic acid (AMC) GDSs superposition methode. **(B)** AT-CZA and AT-AMC double-disc synergy test.

For *S. maltophilia* strains, the DDST demonstrated up to 100% sensitivity in detecting AT-CZA synergy (95% CI, 71 to 100%; Figure 2).

### Synergy confirmation and MIC determination

We found no resistance to the combination of ATA in any *Enterobacterales* strain (Table 3).

**Table 3:** Results of ATA synergy screening (using DDST) and MICs^i^ of AT and ATA combination on the different bacterial strains.

Bacterial strain	Resistance mechanism(s)	Synergy		MIC (μg/ml)			
		DDST	AT GDS MH-AV^ii^	AT GDS MHA	AT GDS MH-AV	AT/CZA GDS MHA	ATA GDS MHA
*Enterobacterales* MBL (*N* = 24)							
*K. pneumoniae*	NDM	Y	Syn	256	0.25	0.25	0.25
*K. pneumoniae*	NDM	Y	Syn	256	0.5	0.75	0.5
*K. pneumoniae*	NDM	Y	Syn	256	0.094	0.25	0.19
*E. coli*	NDM	Y	Syn	256	0.5	0.5	0.75
*E. cloacae*	NDM	Y	Syn	256	0.094	0.25	0.19
*K. pneumoniae*	NDM	Y	Syn	256	0.38	0.38	0.38
*E. cloacae*	NDM	Y	Syn	256	0.125	0.5	0.25
*K. pneumoniae*	NDM	Y	Syn	256	0.25	0.25	0.38
*E. coli*	NDM	Y	Syn	256	1.5	2	1.5
*E. coli*	NDM	Y	Syn	256	0.75	1	1
*K. pneumoniae*	NDM	Y	Syn	256	0.125	0.125	0.094
*K. pneumoniae*	NDM	Y	Syn	48	3	1.5	3
*E. coli*	NDM	Y	Syn	256	1.5	1.5	1.5
*K. pneumoniae*	NDM	Y	Syn	256	0.75	1	0.5
*K. pneumoniae*	NDM	Y	Syn	256	0.25	0.25	0.19
*K. pneumoniae*	NDM	Y	Syn	256	0.38	0.38	0.25
*E. coli*	NDM	Y	Syn	256	3	3	2
*C. freundii*	OXA48 + NDM	Y	Syn	48	0.25	0.5	0.25
*C. freundii*	OXA48 + NDM	Y	Syn	32	0.25	0.75	0.19
*E. Coli*	NDM	Y	Syn	256	3	4	4
*K. pneumoniae*	NDM	Y	Syn	96	0.094	0.064	0.064
*K. pneumoniae*	NDM	Y	Syn	48	0.064	0.064	0.064
*K. pneumoniae*	OXA48 + NDM	Y	Syn	256	0.38	0.5	0.38
*K. pneumoniae*	NDM	Y	Syn	128	0.094	0.064	0.064
*Enterobacterales* SBL (*N* = 18)							
*K. pneumoniae*	KPC	Y	Syn	256	0.38	0.5	0.5
*K. pneumoniae*	OXA48	Y	Syn	256	0.19	0.25	0.19
*K. pneumoniae*	OXA48	Y	Syn	256	0.5	0.75	0.75
*K. pneumoniae*	KPC	Y	Syn	256	0.75	1	0.75
*K. pneumoniae*	OXA48	Y	Syn	256	0.75	1.5	1.5
*K. pneumoniae*	KPC	Y	Syn	256	1.5	1.5	1
*K. Variicola*	KPC	Y	Syn	256	0.75	1.5	1.5
*K. pneumoniae*	OXA48 + KPC	Y	Syn	256	0.75	0.75	1
*K. pneumoniae*	KPC	Y	Syn	256	0.75	0.75	1
*K. pneumoniae*	OXA48	Y	Syn	96	0.25	0.38	0.25
*K. pneumoniae*	KPC	Y	Syn	256	0.38	0.5	0.38
*E. cloacae*	OXA48	Y	Syn	256	0.38	0.5	0.5
*K. pneumoniae*	OXA48	Y	Syn	256	0.75	0.75	0.75
*K. pneumoniae*	KPC	Y	Syn	256	0.38	0.75	0.38
*K. pneumoniae*	OXA48	Y	Syn	256	0.19	0.25	0.25
*K. pneumoniae*	KPC	Y	Syn	256	0.5	0.75	0.75
*K. pneumoniae*	OXA48	Y	Syn	128	0.064	0.094	0.064
*K. pneumoniae*	KPC	Y	Syn	256	0.064	0.125	0.064
*Pseudomonas aeruginosa* (*N* = 12)							
*P. aeruginosa*	VIM	N	Ind	32	32	32	32
*P. aeruginosa*	VIM	Y	Syn	96	24	24	24
*P. aeruginosa*	VIM	N	Ind	32	32	32	24
*P. aeruginosa*	VIM	N	Ind	128	192	96	64
*P. aeruginosa*	VIM	N	Ind	96	48	24	32
*P. aeruginosa*	VIM	N	Ind	32	24	24	24
*P. aeruginosa*	VIM	Y	Syn	64	3	2	4
*P. aeruginosa*	VIM	Y	Ind	64	24	24	24
*P. aeruginosa*	VIM	N	Ind	256	128	64	96
*P. aeruginosa*	VIM	N	Ind	48	24	24	32
*P. aeruginosa*	VIM	Y	Syn	64	2	1.5	2
*P. aeruginosa*	VIM	Y	Ind	32	24	24	24
*Stenotrophomonas maltophilia* (*N* = 11)							
*S. maltophilia*		Y	Syn	256	2	1.5	1.5
*S. maltophilia*		Y	Syn	256	2	2	3
*S. maltophilia*		Y	Syn	256	2	3	3
*S. maltophilia*		Y	Syn	256	2	3	2
*S. maltophilia*		Y	Syn	256	24	24	24
*S. maltophilia*		Y	Syn	256	6	6	6
*S. maltophilia*		Y	Syn	256	12	3	12
*S. maltophilia*		Y	Syn	256	3	3	2
*S. maltophilia*		Y	Syn	256	2	1.5	2
*S. maltophilia*		Y	Syn	256	2	4	2
*S. maltophilia*		Y	Syn	256	2	2	2

AT, aztreonam; ATA, aztreonam/avibactam combination; CZA, ceftazidim-avibactam; DDST, double-disc synergy test; EUCAST, European Committee on Antimicrobial Susceptibility Testing; GDS, gradient diffusion strip; MHA, Mueller Hinton agar; MH-AV, Mueller Hinton agar plate supplemented with avibactam 4 mg/L; N, no; PK/PD, Pharmacokinetics/pharmacodynamics; Y, yes.

i Red, orange, and green colored MICs correspond to resistant, intermediate (susceptible at increased exposure) and susceptible categorization, respectively, according to EUCAST breakpoints (version 13.0) for the tested microorganism. Interpretations of susceptibility to ATA combination was based on AT EUCAST breakpoints for the tested microorganism. PK/PD breakpoints of AT were used to interpret the MIC for *S. maltophilia* as there is no species-specific recommendation for this antibiotic in this species.

ii Synergy between AT and avibactam (using AT GDS applied on MH-AV) was interpreted as follows: - Synergy (Syn) ≥ 2 two-fold dilution decrease in MIC. -Indefference (Ind) < 2 two-fold dilution decrease in MIC.

As expected, all SBL-positive *Enterobacterales* strains (CZA susceptible) were susceptible (17/18, 94%), or susceptible at increased exposure (1/18, 0.06%) to the combination of ATA with MIC50 and MIC90 of, respectively, 0.38 and 0.75 μg/ml.

All MBL-positive *Enterobacterales* strains were found to be susceptible (19/24, 79%) or susceptible at increased exposure (5/24, 21%), with MIC50 and MIC90 of, respectively, 0.25 and 3 μg/ml.

On the other hand, only 17% (2/12) of the VIM positive *P. aeruginosa* strains were found to be susceptible to the combination of ATA at increased exposure, with a MIC50 and MIC90 of, respectively, 24 and 128 μg/ml.

Nine out of 11 (82%) of the *S. maltophilia* strains were susceptible (8/11, 73%), or susceptible at increased exposure (1/11, 9%) to the combination of AT and avibactam, with MIC50 and MIC90 of, respectively, 2 and 12 μg/ml.

### Validation of the GDSs superposition method and the ATA GDS

Only one strain showed a more than 1 two-fold dilution difference between MH-AV and the ATA GDS.

We were able to demonstrate a strong positive correlation with a Spearman correlation coefficient of r_s_ (65) = 0.985, *p* = <0.001 and a PEA of 98%, thereby validating the commercial ATA GDS.

In contrast, the GDSs superposition method showed, compared to MH-AV, a PEA of 87%.

Despite a lower PEA than the commercial ATA GDS, the difference in the occurrence of a ± 1 two-fold dilution between the two methods, compared to the reference method (MH-AV, 4 mg/L), was not significant (*p* = 0.221). The GDSs superposition method still demonstrated a strong correlation with *r_s_* (47) = 0.964, *p* = <0.001 and did not lead to any difference in the interpretation of susceptibility.

## Discussion

A limitation of the current study is the small number of bacterial strains. Only 65 strains were collected, all from patients at a single tertiary healthcare center. Since the rate and mechanisms of resistance vary according to geographical location and type of healthcare facility, the results of the present study may not be extrapolable to other settings. Furthermore, the interpretation of *S. maltophilia* sensitivities is not comparable with other studies given the use of EUCASTs PK/PD breakpoints for AT and CZA in our study instead of the frequently used CLSI breakpoints for *P. aeruginosa* in other studies.

Finally, the use of the MH-AV method as a reference, in the absence of broth microdilution, is another limitation.

In the context of escalating antibiotic resistance, the need for novel therapeutic options cannot be overstated. Following a promising clinical trial with CZA-AT, two further RCTs, REVISIT and ASSEMBLE (awaiting publication), appear to reinforce the effectiveness of the ATA pairing in treating MBL-positive Gram-negative bacterial infections (Clinical Trials.gov, 2023). This underlines the demand for precise synergy detection tools and reliable MIC determination tests (Falcone et al., 2021).

Our research has shown that the DDST is an efficient means of identifying synergy between AT and avibactam. The high negative predictive value of DDSTs enables more accurate selection of GDSs, thereby conserving time and resources. Our ATA GDS demonstrated a strong correlation with the reference method and showed a PEA exceeding 95%, thereby endorsing the ATA GDS. Despite falling short of the anticipated 95% PEA, the CZA-AT superposition method still exhibited a robust correlation. This discrepancy, while noticeable, is statistically insignificant and does not alter the interpretation of susceptibility. Therefore, ATA GDS is the favored option for MIC determination, whereas the superposition method can serve as a substitute for the commercial test.

As predicted, all SBL-positive *Enterobacterales* that are susceptible to CZA are also susceptible to ATA, adding another option to the treatment of SBL-positive *Enterobacterales*. Although both MIC50 and MIC90 seem to favor the latter, its superiority over CZA still needs validation using a time-kill assay. Furthermore, the influence of ATA on the induction of resistance and on the selection pressure of the bacterial flora remains unexplored (Yu et al., 2021).

The majority (79%) of MBL-positive *Enterobacterales* were susceptible, or susceptible at increased exposure (21%), to the ATA combination. This suggests the value of considering ATA combination therapy in infections caused by MBL-positive, Gram-negative bacilli, prior to obtaining MIC results.

Conversely, only 17% of MBL-positive *P. aeruginosa* strains showed susceptibility at increased exposure to the ATA combination. This suggests resistance to AT is not due to the presence of a plasmid-encoded β-lactamase, but rather due to factors like hyperactive efflux systems, impermeability, variations of derepressed *Pseudomonas*-derived cephalosporinases, or OXA enzymes (apart from OXA-48; Sreenivasan et al., 2022). It also warns that *P. aeruginosa* susceptibility interpretations using the superposition method should be approached with caution. A false impression of susceptibility may be made when interpreting the combination of ATA using AT breakpoints, particularly in bacterial strains with a CZA MIC of 16 μg/L. The misinterpretation could be circumvented with DDST, as an absence of a synergy zone between AT and CZA would indicate potential resistance, despite an MIC of 16 μg/L.

The effectiveness of a β-lactamase inhibitor with AT co-administration, is well-known to inhibit L2 and restore activity against L1, is a widely accepted practice in *S. maltophilia* isolates (Calvopiña et al., 2017). Clavulanic acid, demonstrated to have superior activity compared to sulbactam and tazobactam, has justified the pairing of ticarcillin and clavulanic acid since the 90s (Lecso-Bornet and Bergogne-Bérézin, 1997). The combination of AT with clavulanic acid or newer β-lactamase inhibitors like avibactam, relebactam, and vaborbactam have seen renewed interest since the withdrawal of ticarcillin-clavulanic acid from the market in 2015. Our study, like previous research, demonstrates that both avibactam and clavulanic acid, when combined with AT, provide promising therapeutic potential. In our tests, avibactam and clavulanic acid (data not shown) restored AT susceptibility at increased exposure in 91 and 82% of tested *S. maltophilia* strains, respectively (Biagi et al., 2020). Nevertheless, the ATA combination will likely be favored over the AT-clavulanic acid combination due to lower resistance frequency, superior time-kill assay results, and the imminent availability of a fixed drug combination from Pfizer (Clinical Trials.gov, 2023; Biagi et al., 2020).

## Conclusion

Despite its limited efficacy against *P. aeruginosa*, ATA has demonstrated a broad spectrum of activity against multi-resistant *S. maltophilia*, SBL- and MBL-positive *Enterobacterales.*

The DDST is a sensitive tool for the detection of synergy between AT and avibactam.

ATA GDS should be preferred for MIC determination of the ATA combination, while the GDSs superposition method can be used as an alternative to the commercial test.

## Data availability statement

The original contributions presented in the study are included in the article/supplementary material, further inquiries can be directed to the corresponding author.

## Author contributions

GV: Conceptualization, Formal analysis, Investigation, Methodology, Software, Writing – original draft. MN: Writing – review & editing. AS: Writing – review & editing. EH: Data curation, Writing – review & editing. KV: Resources, Writing – review & editing. LV: Formal analysis, Writing – review & editing. MO: Writing – review & editing. KD: Writing – review & editing. TD: Writing – review & editing. DP: Conceptualization, Methodology, Project administration, Supervision, Validation, Writing – review & editing. IW: Formal analysis, Project administration, Supervision, Validation, Writing – review & editing.
